# The Lebanese left ventricular assist device experience, a success story despite the odds

**DOI:** 10.1186/s13019-020-01235-7

**Published:** 2020-07-28

**Authors:** R. Hamdan, S. Fakih, M. Mohammad, F. Charif, H. Abdallah, S. Safa, F. Al Ali, M. Issa, B. Damen, A. el Zein, M. Younes, A. Rabah, M. Saab

**Affiliations:** 1 Cardiology department, Beirut Cardiac Institute, Beirut, Lebanon; 2 Cardiology fellow, Cardiology department, Beirut Cardiac Institute, Beirut, Lebanon; 3 Lebanese Society of Cardiology, Cardiology department, Bahman Hospital, Beirut, Lebanon; 4 Critical care unit, Beirut Cardiac Institute, Beirut, Lebanon; 5 Cockrell school of engineering, University of Texas, Austin, TX USA; 6 Cardiac surgeon, Cardiac Surgery department, Beirut Cardiac Institute, Beirut, Lebanon; 7 Cardiac Surgery department, Beirut Cardiac Institute, Beirut, Lebanon

**Keywords:** Left ventricular assist devices, Middle East, Mortality, Morbidity

## Abstract

**Background and aim:**

Heart failure is still a leading cause of mortality and morbidity. Assist devices are reserved for advanced heart failure patients with no other therapeutic options.

We aim in this paper to describe the characteristics and outcome of Lebanese left ventricular assist device (LVAD) patients.

**Results:**

From 2010 till December 2019, 78 patients were implanted with assist devices at the Beirut cardiac Institute, 82 pumps were used. To the most recent follow up after 10 years, 26 patients died (34%). 24 patients of 35 (68%) survived more than 5 years. Seven patients only (9%) died during one month of surgery. One year mortality was 19% (15 patients). The leading cause of early mortality was infection, whereas cerebrovascular accidents CVA were the leading cause of late mortality. Pump thrombosis occurred in 12% of the cases. The most serious long term complication was haemorrhagic CVA. Only seven patients (9%) received heart transplantation, with a mean time on support prior to transplantation of 1303 ± 213 days.

**Conclusion:**

In this manuscript we reported the characteristics and outcome of the largest population of LVAD patients in Lebanon. The survival rate was 81% at one year. These findings were comparable to the international registries except for rates of heart transplantation. More efforts should be made to encourage organ donation in Lebanon.

## Introduction

Heart failure mortality and morbidity remains very high. Therapeutic modalities in heart failure witnessed a dramatic progression in the last decades starting from advances in medical therapy, to assist devices and heart transplantation [1]. Assist devices are reserved for advanced heart failure patients with no other therapeutic options [1, 2]. The advances in assist devices technique through the time have significantly improved the outcome of heart failure patients. In Lebanon we encounter three majors challenges for this therapy beside the complications that are: the cost, the delayed referral of patients to advanced hear failure units, and the lack of organ donation.

We aim in this paper to describe the characteristics and outcome of LVAD patients in the Beirut Cardiac Institute center, that is the main local institute that has the largest experience in LVAD in Lebanon.

## Material and methods

We retrospectively analysed the data from the patients’ records of heart failure patients who underwent LVAD implantation in our institution (Beirut Cardiac Institute) from 2010 till 2019. Our institution is a regional leader in applying LVAD therapies performing around 60% of lebanese VAD surgeries.

We reported patients’ demographics and device type, heart transplantation, survival, and adverse events.

We used the 3 FDA (food and drug administration) approved VAD devices, Heartmate II, Heartware, and Heartmate III. When we started in 2010, we started with Heartmate II since it was the only FDA approved device, then we introduced Heartware in 2012 to our practice after it got the FDA approval, and finally in 2018 we also started with the newest generation Heartmate III pump.

### Definitions

-Pump thrombosis was defined according to the INTERMACS (Inter Agency Registry for Mechanically Assisted Circulatory Support) definition [2] by: Alterations in pump parameters defined as unexpected power increase with higher than expected flow estimation (“pseudo -flow”) or a precipitous drop in flow and power and/or increase in biochemical markers of hemolysis, including LDH > 2 times the upper limit, or (plasma free hemoglobin) pfHgb > 40 mg/ dl or hemoglobinuria and /or Visualization of organized fibrin in the pump housing after exchange of the LVAD and /or abnormal pump sounds identified by auscultation.

-Right ventricular (RV) failure was defined according to the INTERMACS as well [3] by: signs and symptoms of persistent RV dysfunction characterized by both of the following:

- Documentation of elevated central venous pressure (CVP) by:
  - o Central venous pressure (CVP) or right atrial pressure (RAP) > 16 mmHg.
- Manifestations of elevated central venous pressure characterized by:
  - o Clinical findings of peripheral edema or / ascites/ or palpable hepatomgegalyor
  - o Laboratory evidence of worsening hepatic (total bilirubin > 2.0 mg/dl) or renal dysfunction (creatinine > 2.0 mg/dl).

-Cerebrovascular accident (CVA) was defined by new onset neurological symptoms and imaging confirmation by computed tomography scan of acute haemorrhage and/or ischemia.

-Driveline infection was defined by oozing at the driveline site with documented bacterial cultures.

-Anticoagulation protocol: all patients were started on intravenous unfractionated Heparine, anti vitamin K and Aspirin 100 mg on day one post surgery, we target an INR level between 2 and 3.

### Statistical analysis

Quantitative data was expressed as mean ± standard deviation. Qualitative values were Expressed as percentages. Comparison between quantitative variables was realized via the Welch Two Sample t -test with a significant *p* Value < 0.05.

## Results

**Fig. 1:**
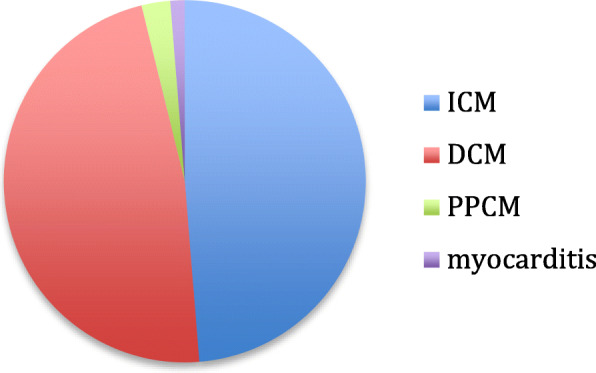
Etiology of heart failure: ICM: ischemic cardiomyopathy, DCM: dilated cardiomyopathy, PPCM: peripartum cardiomyopathy

**Fig. 2:**
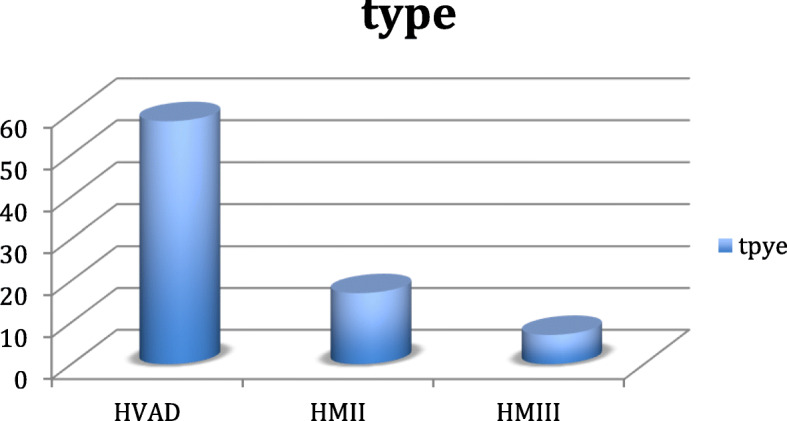
Pump type: HM II (17) 21% HM III (7) 8% HVAD (58) 71%

We established the assist device program in 2010. From 2010 till December 2019, 78 patients were implanted with assist devices at Beirut Cardiac Institute, with a total of 82 pumps used as 4 patients required pump exchange. The baseline characteristics of the patients are shown in Table 1. The mean INERMACS profile was 2.0, 14% of the patients were in INTERMACS profile 4 and 86% were in INTERMACS 1 -3. We did not implant any patients in INTERMACS profile 5, 6, and 7. Forty nine percent of the patients had ischemic cardiomyopathy; the aetiology of heart failure is shown in Fig. 1. Thirty two percent of the patients had concomitant tricuspid plasty during the LVAD surgery, other concomitant surgery are detailed in Table 2. We implanted Heartmate II, Heartware and HeartMate III pumps, the type of pump implanted is shown in Fig. 2.

**Table 1:** Baseline characteristics

Mean age (years)	49.8 ± 12.7
Female	(25) 32%
Diabetes	(32) 41%
Hypertension	(32) 41%
Dyslipidemia	(34) 44%
Smoking	(25) 32%
Body mass index (Kg/m^2^)	24.9 ± 3.1
Previous CABG	(11) 14%
Creatinine	1.4 ± 0.4
ICD/CRTD	(65) 83%
Mean LVEF (%)	18 ± 2.8
LVEDD (mm)	73 ± 5.1
TAPSE (mm)	14.9 ± 3.9
CVP (mmHg)	13.7 ± 4.0
Cardiac index (l/mn/m^2^)	1.7 ± 0.3
INTERMACS	2.0 ± 0.4
BTT	(41) 53%
DT	(33) 42%
BTR	(4) 5%
ECMO	(9) 11.6%
IABP	(4) 5%

**Table 2:** Concomitant surgeries

Tricuspid plasty	(25) 32%
AVR	(2) 3%
Aortic valve plasty	(5) 6%
FOP closure	(1) 1%
CABG (RCA)	(1) 1%
Angioplasty (RCA)	(3) 4%

The VAD implantation technique was performed via mid sternotomy on cardiopulmonary bypass, with beating heart. The left ventricular apex was cored after the sewing ring of the inflow canula was placed at the apex and fixed by 16 to 20 separated stiches. Once the pump is correctly connected to the ring, a manoeuver of de -airing was performed. The out flow graft was anastomosed to the ascending aorta using a continuous suture of prolene 4/0. A polytetrafluoroethylene (PTFE) membrane was used to close the pericardium and cover the pump systematically at the end of the procedure.

Fifty three percent (53%) of our patients were implanted as bridge to transplantation, 42% as destination therapy and 5% as bridge to recovery.

After 10 years follow up, 26 patients died (34%), the mean days of support prior to death was 653 ± 90 days. Seven patients (9%) died during one month of surgery. One year mortality was 19% (15 patients), 5 patients (6%) died of non cardiac death: one died at seven years of support from dementia, 2 patients had brain trauma, and 2 patients died from neoplasia. The first cause of early death was infection, whereas the first cause of late death was CVA, the cause of death is detailed in Table 3.

**Table 3:** Cause of death

	Early death(<one month)	Late death	All death
	(7) 9%	(14) 18%	(26) 33%
Infection and sepsis	(5) 72%	(4) 29%	(9) 35%
CVA	0	(8) 57%	(8) 31%
Ischemic CVA	0	(2) 14%	
Hemorrhagic CVA	0	(6) 43%	
RV Failure	(2) 28%	(1) 7%	(3) 11%
Pump thrombosis	0	(1) 7%	(1) 4%
Non cardiac or device related			(5) 19%

**Fig. 3:**
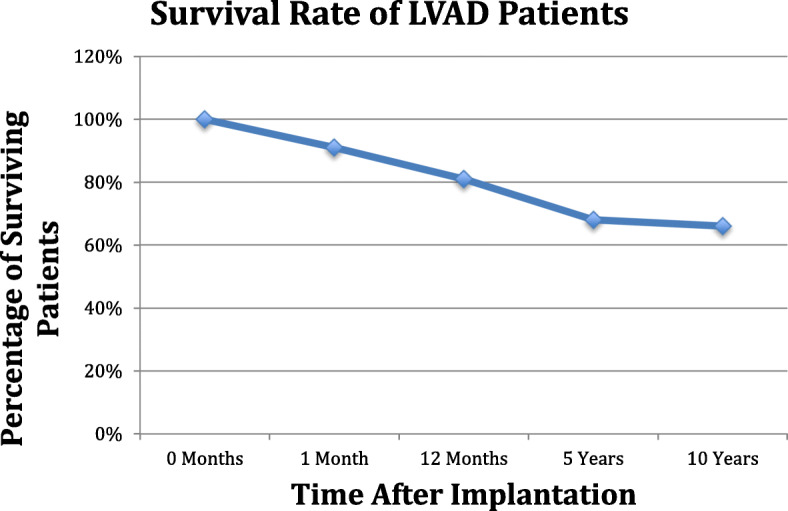
Patient’s survival

From 2010 till 2014, 24 patients of 35 (68%) survived more than 5 years. The survival is presented in Fig. 3. As we showed in Table 1, mean INTERMACS profile was 2.0, we did not implant at INTERMACS 5,6, and 7. None of INTERMACS 4 patients died.

Seven patients (9%) had heart transplantation, with a mean time on support prior to transplantation of 1303 ± 213 days. We found that length of in hospital stay prior to VAD implantation was an independent predictor of death (Table 4).

**Table 4:** Length of in hospital stay prior to LVAD

Mean length (days)	Mean length for surviving patients (days)	Mean length for deceased patients (days)	P value
11.4±	8.4 ± 7.6	16.6 ± 11.7	0.0005

Interestingly we had 2.5% of full recovery (2 patients), both with peripartum cardiomyopathy, we were able to successfully explant the pump and they are both doing fine.

Adverse events are shown in Table 5.

**Table 5:** Adverse events

Pump thrombosis requiring thrombolysis	(10) 12%
CVA ischemic	(5) 6%
CVA hemorrhagic	(11) 14%
Gastrointestinal bleeding	(15) 19%
Severe aortic regurgitation	(1) 1%
Moderate aortic regurgitation	(20) 25%
Driveline infection	(22) 28%
Pump exchange	(4) 5%
Pump malfunction	(1) 1%
Dialysis	(3) 4%
RVF	(7) 9%
ECMO for RVF	(2) 3%

Pump thrombosis occurred in 12% of the patients (10 patients), all cases were systematically treated with Actilyse with a minimum dose of 50 mg and a maximal dose of 100 mg, according to the regression of the thrombosis parameters, mainly the high watt/high output. The success rate of thrombolysis was 90%, we had to exchange the pump in 20% of the cases (2 patients) one for Actilyse failure and the second for recurrent thrombosis (more than 6 times) requiring Actilyse each time.

## Discussion

Although it is a retrospective single center registry, we consider this population a representative sample of the lebanese population since our center is the main local institute that has the largest experience in LVAD in Lebanon and the patients are dispatched from all the lebanese regions. The outcome of this population was similar to the IMACS (International Registry for Mechanically Assisted Circulatory Support) data [4]. Among all patients in the IMACS Registry, the 12 -month survival estimate was 80%, with 75% survival at 18 months [4].

In another report the four -year survival approximates 60% reference [5]. We had a 5 years survival of 68%, this was achieved despite the fact that most of the patients were in INTERMACS stages 1, 2and 3. We interestingly found a correlation between duration of in hospital stay prior to LVAD implantation and mortality, the cause of the delay was not reported in all cases, but mainly consisted of infection or sadly due the financial barrier and to the delay in acquiring the financial approval for implantation. Only 2 of the national healthcare providers in Lebanon cover the LVAD procedure costs: the national social security fund and the lebanese army fund, this roughly represents around 40% only of lebanese the population. As mentioned earlier the other barrier to VAD therapy in Lebanon is the delayed referral of patients in advanced heart failure to specialized centres, continuous efforts are being done by the Lebanese Society of Cardiology, and buy our team at the Beirut Cardiac Institute to raise awareness in this field in order to improve outcome. We are organizing regularly national advanced heart failure conferences and workshops, targeting all the lebanese cardiologists to improve patient referral rate and encourage organ donation.

The likelihood of receiving a heart transplant within 6 months was estimated to be 14% among patients actively listed for transplant at the time of LVAD implant [4], unfortunately compared to this data we were severely below the reported numbers, wich is due again to the lack of education and awareness. The shortage of organ donation enabled us to witness very long support duration, and the transformation of patients initially implanted as bridge to transplantation (BTT) to destination therapy (DT), 3 patients are still on support since 10 years.

We interestingly had full recovery in 2 patients, both had PPCM, after a thorough weaning protocol according to Dandel et al. [6] LVADs were successfully explanted via sternotomy, with both patients doing well and maintained on medical therapy with mildly reduced ejection fraction, (45 and 48% respectively). To the best of our knowledge these were the first cases of LVAD explantation for left ventricular recovery in the Middle East area.

We had relatively a low incidence of RV failure post VAD (9%), recent data reported that 20 % or more of patients undergoing isolated LVAD implantation experienced RV failure [7], which is a leading cause of premature morbidity and mortality. From a hemodynamic perspective, activation of an LVAD increases venous return, potentially overwhelming a functionally impaired RV, leading to RV dilatation, tricuspid regurgitation, leftward shift of the interventricular septum, and decline in RV stroke volume [7].

In our protocol we use phosphodiesterase type 5 inhibitors such as sildenafil that might help to prevent and treat RV failure and pulmonary hypertension ([7, 8] although recent data found controversial results regarding the use of Sildenafil, as adjunctive treatment of post LVAD RV dysfunction was not associated with improved clinical outcomes [9].

Around 12 % of our patients required ECMO prior to VAD implantation, all of these patients were in INTERMACS 1 profile. It has been showed recently that INTERMACS 1 patients who benefited from VA ECMO had a significant reduction in the risk of death compared with INTERMACS 1 non VA ECMO group (*p* = 0.035) [10].

We had better outcome in terms of mortality for INTERMACS 4 patients (0 death). In addition we did not implant VAD for INTERMACS 5, 6 and 7 patients, this was due to many factors, first the difficult financial coverage for the patients limit us to choose the most urgent ones, second more stable patients are always more reticent to VAD therapy, third and most importantly, the lack of current data supporting VAD implantation for this patients entity. This is supported by the results of the ROADMAP [11] study that showed significant benefits of LVAD vs optimal medical therapy in INTERMACS 4 patients and not in IM5 -7 patients.

Gastrointestinal bleeding occurred in 32% of the patients in the IMACS report [4]. The lower peripheral vascular pulsatility may promote arteriovenous malformations, that amplify the bleeding risk [12]. Arteriovenous malformation is the most common etiology for gastrointestinal bleeding in our population in concordance with the known data reference [13].

Thrombosis is a relatively frequent and serious adverse event with a reported incidence of 5.5 to 12.2% in patients with MCS [14, 15]. Thrombosis is associated with significant morbidity. Systemic rtPA is the most commonly used and effective medical therapy, in case of failure of thrombolysis, pump exchange should be the next step [14.15].

In our experience we had 12% of pump thrombosis that necessitated thrombolysis with rtPA (Actilyse), with a dose between 50 and 100 mg. We performed pump exchange for pump thrombosis in 2 patients only, and one patient only died of recurrent pump thrombosis after exchange. Factors that may contribute to thrombus formation are subtherapeutic anticoagulation, low pump speed, and elevated blood pressure [16].

Cerebrovascular events were the most limiting and life threatening adverse event in our experience, occurring in 20% of patients (6% had ischemic CVA, and 14% had hemorrhagic CVA) and was responsible of 31% of the mortality. Increased afterload decreases pump flow and increases the risk of neurological events and end -organ damage [16].

Titration of medical therapy to maintain a mean arterial blood pressure in the normal range is imperative to optimize forward flow and to prevent adverse events. Neurohormone -modifying agents such as angiotensin -converting enzyme inhibitors, angiotensin receptor blockers, β -blockers, and mineralocorticoid receptor antagonists are used to decrease afterload, to improve pump function [16].

The Endurance trial confirmed that BP management is associated with reduced stroke rates in HVAD subjects [17].

Aortic insufficiency is known to complicate approximately 25% of patients with continuous flow assist devices, we had the same findings, with only one patient having severe symptomatic aortic regurgitation (AR), she is on support since 8 years and she is a priority for heart transplantation. Aortic insufficiency after mechanical circulatory support remains not fully understood, however, continuous closure of the aortic valve is thought to be a central factor [16].

Due to the discrepancy of the sample sizes of each pump we did not relate the outcome according to the pump type. Comparison between pumps was analysed in the MOMENTUM 3 trial [18] that showed significantly less pump thrombosis event with centrifugal compared to axial pumps (1.1 versus 15.7%).

Centrifugal flow outperforms axial flow recipients in regards to GI bleeding and freedom from hemocompatibility related adverse events. No significant difference in the actuarial freedom from all strokes or either stroke subtype (hemorrhagic or ischemic) was seen among the two types of pumps [5].

## Conclusion

In this manuscript we reported the characteristics and outcome of the largest population of LVAD patients in Lebanon. The survival rate was 81% at one year. Pump thrombosis occurred in 12% of the cases. The most serious long term complication was haemorrhagic CVA. These findings were comparable to the international registries except for the likelihood of heart transplantation. More efforts should be taken to encourage organ donation in Lebanon.

### Limitations

Our main limitation was the relatively low number of patients and the retrospective analysis, which prompt this study to be a descriptive analysis only, any correlation or specific risk factor establishment for short and long term mortality perdictors would be of low statistical significance.

## Supplementary information

**Additional file 1.**

## Data Availability

Supporting data is attached as supplemental material.

## Abbreviations

DCM: Dilated cardiomyopathy
ECMO: Extracorporeal membrane oxygenation
ICM: Ischemic cardiomyopathy
INTERMACS: Inter agency registry for mechanically assisted circulatory support
IMACS: International Registry for Mechanically Assisted Circulatory Support
LDH: Lactate dehydrogenase
LVAD: Left ventricular assisted device
RAP: Right atrial pressure
AR: Aortic regurgitation
CVA: Cerebral vascular accident
CVP: Central venous pressure
Pfhb: Plasma free hemoglobin
PPCM: Peripartum cardiomyopathy
VAD: Ventricular assisted device
Rt pa: Recombinant tissue plasminogen activator
RV: Right ventricle
CABG: Coronary artery bypass grafting
IABP: Intra aortic balloon pump
INR: International normalized ratio
FDA: Food and drug administration
PTFE: Polytetrafluoroethylene
